# Effect of Different Exercise Methods on Non-Alcoholic Fatty Liver Disease: A Meta-Analysis and Meta-Regression

**DOI:** 10.3390/ijerph18063242

**Published:** 2021-03-21

**Authors:** Yingzhe Xiong, Qingwen Peng, Chunmei Cao, Zujie Xu, Bing Zhang

**Affiliations:** 1 Division of Sports Science and Physical Education, Tsinghua University, Beijing 100081, China; xyz19@mails.tsinghua.edu.cn (Y.X.); caocm@tsinghua.edu.cn (C.C.); xuzj20@mails.tsinghua.edu.cn (Z.X.); 2 School of Kinesiology and Health, Huaihua University, Huaihua 418000, China; pqw@hhtc.edu.cn

**Keywords:** exercises, non-alcoholic fatty liver disease, randomized clinical trials, meta-analysis

## Abstract

Exercise could alleviate non-alcoholic fatty liver disease (NAFLD), but it was not clear which exercise methods could effectively treat NAFLD. The purpose of this systematic review and meta-analysis was to evaluate the effects of different exercise patterns on eight indicators in patients with NAFLD. We searched PubMed, Scopus, Web of Science, China National Knowledge Infrastructure, and the Wanfang Data from its inception to 30 June 2020. This review includes all randomized controlled trials (RCT) that assessed and compared the effects of different exercise on eight indicator parameters in patients with NAFLD. The results indicate that aerobic exercises could significantly improve the eight indicators in patients with NAFLD including triglycerides (TG, weighted mean difference (WMD) = −0.53, 95%CI: −0.68~−0.39, Z = 7.37, *p* < 0.01), total cholesterol (TC, WMD = −0.39, 95%CI: −0.55~−0.23, Z = 4.76, *p* < 0.01), low density lipoprotein (LDL, WMD = −0.47, 95%CI: −0.68~−0.26, Z = 4.33, *p* < 0.01), high density lipoprotein (HDL, WMD = 0.12, 95%CI: 0.05~0.18, Z = 3.56, *p* < 0.01), alanine aminotransferase (ALT, WMD = −6.14, 95%CI: −10.99~−1.29, Z = 2.48, *p* < 0.05), aspartate aminotransferase (AST, WMD = −5.73, 95%CI: −9.08~−2.38, Z = 3.36, *p* < 0.01), and body mass index (BMI, WMD = −0.85, 95%CI: −1.19~−0.51, Z = 4.92, *p* < 0.01). Resistance exercises could significantly reduce the levels of TG (WMD = −0.56, 95%CI: −0.85~−0.28, Z = 3.86, *p* < 0.01) and AST (WMD = −2.58, 95%CI: −4.79~−0.36, Z = 2.28, *p* < 0.05) in the patients. High-intensity interval training could significantly improve the level of ALT (WMD = −6.20, 95%CI: −9.34~−3.06, Z = 3.87, *p* < 0.01) in patients with NAFLD. These three exercise methods had different effects on the eight indexes of NAFLD in our present meta-analysis, providing some reference for the establishment of exercise prescription for patients with NAFLD.

## 1. Introduction

Non-alcoholic fatty liver disease (NAFLD) refers to a clinical and pathological syndrome characterized by excessive fat deposits in hepatocytes that result from causes other than alcohol and liver damage factors. It is an acquired metabolic stress injury associated with insulin resistance and genetic susceptibility [1,2]. With the booming economy and rapid changes in lifestyles, the development of NAFLD has become a major public health problem, especially in China. As of 2018, the prevalence of NAFLD in China reached 32.9% [3]. It was estimated that by 2030, the total number of patients with NAFLD in China will be more than 300 million with the fastest growth rate around the world. By then, NAFLD will be the largest burden in the control and prevention of chronic diseases [4].

Currently, NAFLD is an underestimated health problem threatening peoples’ lives. Apart from inducing liver diseases, it might also lead to or accelerate the development of cardiovascular and metabolic diseases. The pathogenesis of NAFLD is complicated, and genetic susceptibility and metabolic disorders are considered as potential causes [5]. Due to the mysterious pathogenesis and individual heterogeneity, no specific drug has been developed for the treatment of NAFLD. Since lifestyle changes are able to reduce levels of liver enzymes and improve fatty livers, health education and lifestyle intervention are recommended as the first-line treatment for NFALD [6,7]. A previous study revealed that exercise could effectively reduce levels of liver fat and serum alanine aminotransferase (ALT), which is an efficacious treatment for NAFLD with low expense and high cost-effectiveness [8]. In addition, Hallsworth et al. found that high-intensity interval training (HIIT) could significantly reduce levels of liver fat, serum fat, ALT, and aspartate aminotransferase (AST) in patients with NAFLD [9]. Moreover, aerobic exercises were shown to have therapeutic effects on NAFLD by reducing serum triglycerides and low density lipoproteins [10].

Although multiple clinical studies have been carried out to evaluate the efficacy of exercises in the treatment of NAFLD, there was no consistent conclusion because of the variation of exercise methods, detection indicators, and sample sizes of these studies [11,12]. Therefore, a systematic review and analysis were required to determine the efficacy of different exercise methods in the treatment of NAFLD. In this study, we reviewed multiple randomized clinical trials (RCTs) exploring the efficacy of different exercise methods in NAFLD and analyzed their characteristics using meta-analysis, aiming to provide a scientific and comprehensive reference for making exercise prescriptions in the treatment of NAFLD.

## 2. Materials and Methods

### 2.1. Search Strategies

The literature search was performed in PubMed, Scopus, Web of Science, China National Knowledge Infrastructure, and the Wanfang Data from January 2000 to April 2020. The search keywords were “exercise”, “training”, “nonalcoholic fatty liver”, “fatty liver”, and “steatohepatitis”, and only randomized controlled trials were included in the present meta-analysis.

### 2.2. Inclusion and Exclusion Criteria

Studies were included according to the following criteria: (1) RCTs with exercises as the intervention treating patients with NAFLD; (2) research subjects were diagnosed as having NAFLD through pathological or imaging examinations; (3) no significant difference was detected between the experimental group and the control group before the intervention; (4) there was an exercise intervention in the experimental group compared with the control group, and the intervention time was over 8 weeks; (5) the detection indicators included triglycerides (TG), total cholesterol (TC), low density lipoprotein (LDL), high density lipoprotein (HDL), ALT, AST, γ-glutamyl transpeptidase (GGT), and body mass index (BMI); (6) the relevant data before and after the intervention could be obtained. Studies were excluded based on the following criteria: (1) studies such as animal experiments, abstracts, case reports, reviews, systematic reviews, and repeated publications; (2) the duration of exercise intervention with less than 8 weeks; (3) the detection indicators did not meet the inclusion criteria; (4) unable to obtain required data.

### 2.3. Data Extraction

The study selection and data extraction were performed by two authors independently (Yingzhe Xiong and Qingwen Peng). According to the above exclusion conditions, the unqualified literature could be excluded by reading the abstract or the full text. The following data were extracted from the included studies: the name of the first author, publication year, intervention arms, sample size, gender, age, intervention methods (exercise methods, intensity, frequency, and duration), diet, medication, and detection indicators.

### 2.4. Quality Assessment

The quality of included studies was assessed by the RCT quality evaluation method according to the Cochrane Handbook for Systematic Reviews of Interventions. The evaluation was performed regarding randomization methods, allocation concealment, blinding of patients and physicians, outcome evaluations, the integrality of data, selective reporting, and other sources of bias [13].

### 2.5. Statistical Analysis

Review Manage 5.4 and Stata 16.0 were used to make a Cochrane bias risk assessment diagram and statistical analysis. Meta-analysis was conducted in accordance with the Preferred Reporting Items for Systematic Review and Meta-Analysis (PRISMA) guidelines [14]. All experimental data were continuous variables. The value of quantitative data was shown by the weighted mean difference (WMD) with 95% confidence interval (CI). The statistics of the heterogeneity test obey the Gaussian distribution. The significance level was α = 0.05, and *p* < 0.05 reflected a heterogeneity among the studies. In addition, the heterogeneity among studies was assessed by the I^2^ test with the significance level of 40%. I^2^ > 40% represented a large heterogeneity between studies. The combined effect analysis of non-heterogeneous studies was conducted by the fixed effects model, whereas the random effects model was adopted for heterogeneous studies. The source of heterogeneity was analyzed by subgroup analysis in Review Manage 5.4. Sensitivity analysis and univariate meta regression analysis were analyzed in Stata 16.0. Meta regression analysis was adopted to explore the source of heterogeneity by analyzing the publication year, sample size, intervention time, diet, and medication of each study. Egger’s tests were conducted to assess the potential publication bias when there were more than 10 studies included.

## 3. Results

### 3.1. Literature Retrieval

**Figure 1:**
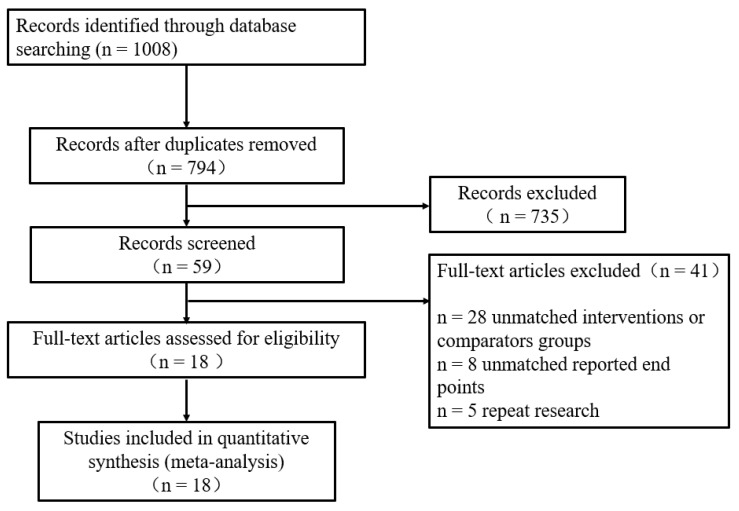
Flow diagram of the selected process for the included studies.

The flowchart of literature retrieval is shown in Figure 1. A total of 1008 studies were identified from the electronic databases. Then, 794 studies remained after excluding 214 duplicates, and 59 studies were found to be potentially relevant. After reviewing the full text, 41 studies were excluded according to the exclusion criteria. Finally, 18 studies were included in the present meta-analysis.

### 3.2. Basic Characteristics of Included Studies

A total of 1250 patients in 18 studies were included for further analysis. The age of these patients ranged from 32 to 70 years old. In these studies, two adopted a HIIT intervention [9,11]; 15 adopted aerobic exercise [10,11,12,15,16,17,18,19,20,21,22,23,24,25,26]; one adopted aerobic exercise combined with resistance exercise [27]; and three adopted resistance exercise [12,18,28]. The basic characteristics of the included studies including the name of the first author, publication year, intervention arms, sample size, gender, age, intervention methods (exercise methods, intensity, frequency, and duration), diet, medication, and detection indicators are summarized in Table 1.

### 3.3. Quality Assessment of Included Studies

**Figure 2:**
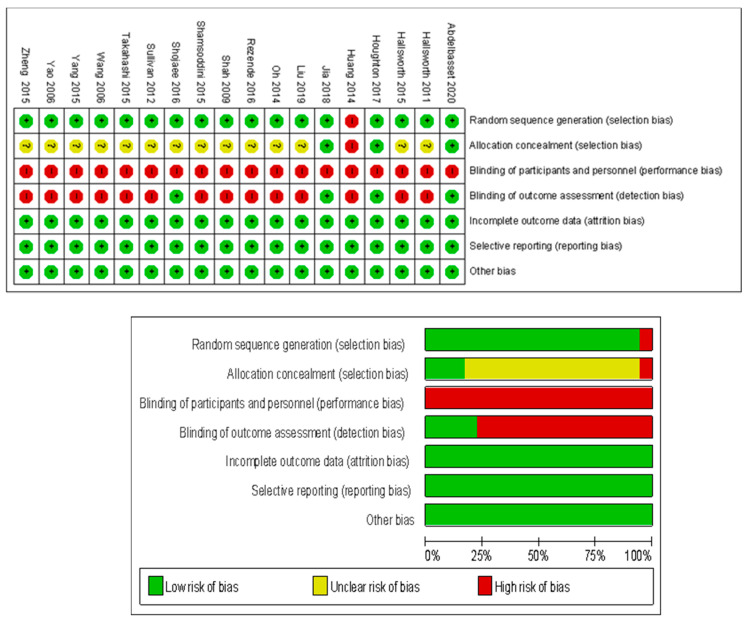
The Cochrane bias risk assessment diagram for the included research.

The quality of included studies was assessed by the RCT quality evaluation method according to the Cochrane Handbook for Systematic Reviews of Interventions. The Cochrane bias risk evaluation diagram shows the risks of different biases of the 18 included studies. The blinding of participants and personnel (performance bias) and outcome assessment (detection bias) exhibited the highest risk in the included studies whereas selection biases exhibited moderate risks. In addition, other biases such as attribution bias and reporting bias exhibited low risks, see Figure 2.

### 3.4. Meta-Analysis and Publication Bias Evaluation

#### 3.4.1. Meta-Analysis and Publication Bias Evaluation of TG

**Figure 3:**
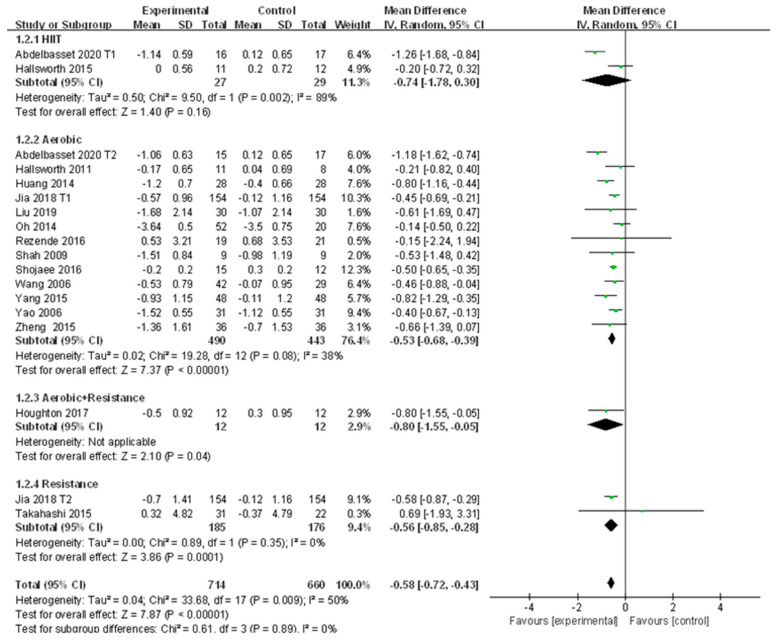
Subgroup analysis of different exercise styles on triglycerides (TG).

A total of 16 studies reported the change of TG in patients with NAFLD before and after exercise intervention. Moderate heterogeneity was detected among the studies (I^2^ = 50%, *p* < 0.01). The random effects model revealed that the level of TG in patients with NAFLD was significantly reduced after exercise intervention compared to that of the control group (WMD = −0.58, 95%CI: −0.72~−0.43, Z = 7.87, *p* < 0.01). Subgroup analysis showed that aerobic exercise could significantly reduce TG in patients with NAFLD (WMD = −0.53, 95%CI: −0.68~−0.39, Z = 7.37, *p* < 0.01), and no heterogeneity was detected in aerobic exercise subgroups (I^2^ = 38%, *p* > 0.05); resistance exercise could significantly decrease TG in patients with NAFLD (WMD = −0.56, 95%CI: −0.85~−0.28, Z = 3.86, *p* < 0.01), and no heterogeneity was detected in resistance exercise subgroups (I^2^ = 0.0%, *p* > 0.05) (Figure 3).

Sensitivity analysis shows that the sensitivity of the included literature is low, indicating that the results of each study are stable and reliable (Supplementary Materials Figure S1). In meta regression analysis, publication year was found to significantly affect the heterogeneity of included studies (*p* < 0.05), whereas the other four factors did not exhibit significant impacts (*p* > 0.05) (Table 2). Therefore, the publication year was the key factor affecting the heterogeneity of studies. Publication bias did not exist in 16 included studies (t = −0.40, *p* > 0.05).

#### 3.4.2. Meta-Analysis and Publication Bias Evaluation of TC

**Figure 4:**
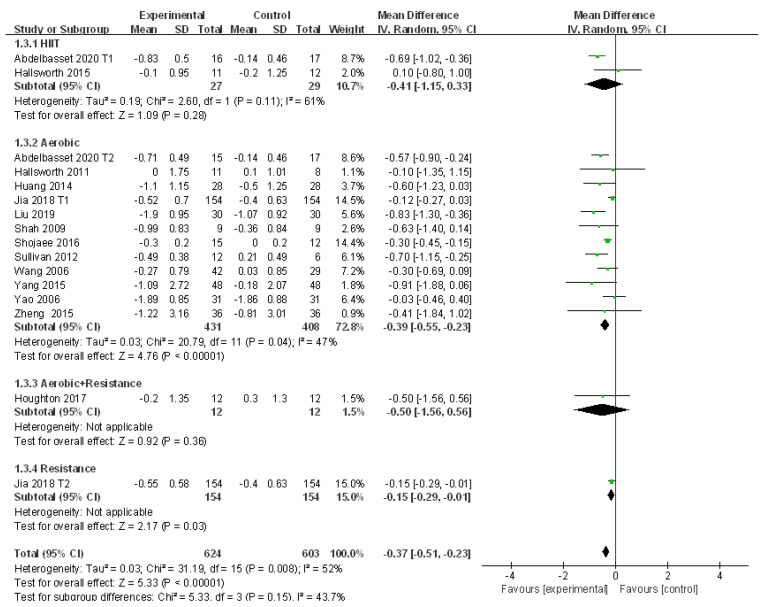
Subgroup analysis of different exercise styles on total cholesterol (TC).

Fourteen studies revealed the change of TC in patients with NAFLD who received exercise intervention. There were large heterogeneities detected among the studies (I^2^ = 52%, *p* < 0.01). The random effects model was applied for meta-analysis, and it revealed that the level of TC in patients with NAFLD was significantly decreased after exercise intervention compared to that of the control group (WMD = −0.37, 95%CI: −0.51~−0.23, Z = 5.33, *p* < 0.01). Subgroup analysis revealed that aerobic exercise could significantly reduce TC in patients with NAFLD (WMD = −0.39, 95%CI: −0.55~−0.23, Z = 4.76, *p* < 0.01) with low heterogeneities among the included studies (I^2^ = 47%, *p* < 0.05) (Figure 4).

The source of heterogeneity from the included studies was explored by the sensitivity analysis. Results showed that the low sensitivity of the included literature had little effect on heterogeneity (Figure S2). In addition, meta regression analysis was conducted to investigate further causes. Publication year was found to be the key factor affecting the heterogeneity (*p* < 0.05), whereas the other factors did not exhibit significant impacts (*p* > 0.05). Therefore, the publication year was the major factor affecting the heterogeneity of studies (Table 3). In addition, Egger’s test revealed that publication bias existed in 14 of the included studies (t = −2.20, *p* = 0.05).

#### 3.4.3. Meta-Analysis and Publication Bias Evaluation of LDL

**Figure 5:**
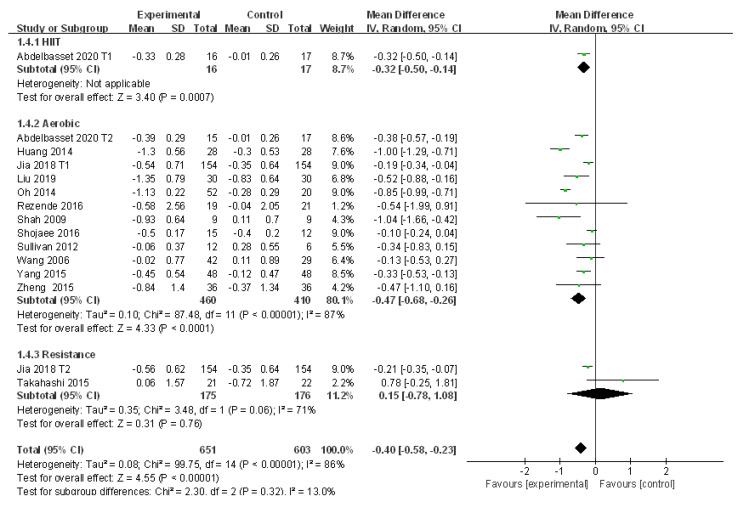
Subgroup analysis of different exercise styles on low density lipoprotein (LDL).

A total of 13 studies explored the efficacy of exercise intervention by evaluating the change of LDL in patients with NAFLD with large heterogeneities (I^2^ = 86%, *p* < 0.01). Meta-analysis was performed with the application of the random effects model, and the source of heterogeneity was analyzed by subgroup analysis, sensitivity analysis, and meta regression analysis. Compared to the control group, LDL was significantly reduced after exercise intervention in patients with NAFLD (WMD = −0.40, 95%CI: −0.58~−0.23, Z = 4.55, *p* < 0.01). Subgroup analysis showed that aerobic exercise could significantly reduce LDL in patients with NAFLD (WMD = −0.47, 95%CI: −0.68~−0.26, Z = 4.33, *p* < 0.01) with significant heterogeneities among subgroups (I^2^ = 87%, *p* < 0.01). In addition, resistance exercise tended to increase LDL in patients with NAFLD but with no significant difference (WMD = 0.15, 95%CI: −0.78~1.08, Z = 0.31, *p* > 0.05), and large heterogeneities were detected in resistance exercise subgroups (I^2^ = 71%, *p* > 0.05) (Figure 5).

The sensitivity analysis showed that excluding several articles could slightly affect the heterogeneity of 13 of the included studies (Figure S3). Meta regression analysis was applied to investigate the effect of the publication year, sample size, intervention time, diet, and medication on the source of heterogeneity, in which diet was found to markedly affect the heterogeneity (*p* < 0.01) (Table 4). In addition, no publication bias was detected in 13 of the included studies (t = −0.28, *p* > 0.05).

#### 3.4.4. Meta-Analysis and Publication Bias Evaluation of HDL

**Figure 6:**
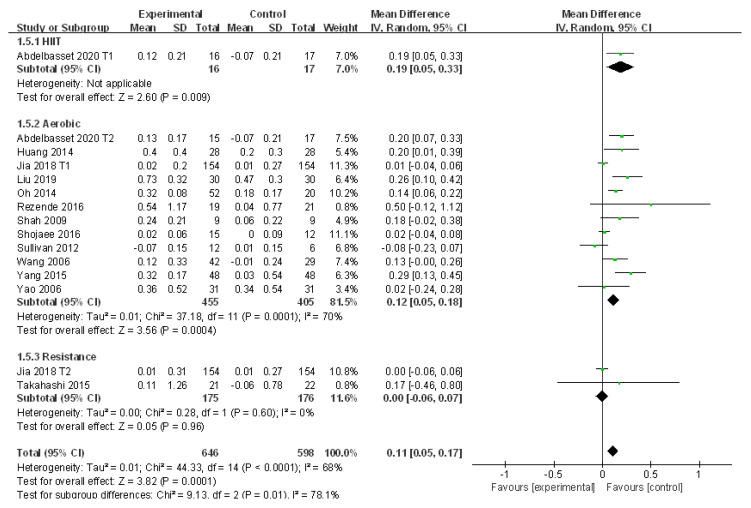
Subgroup analysis of different exercise styles on high density lipoprotein (HDL).

Thirteen studies evaluated the alteration of HDL in patients with NAFLD who received exercise intervention. Large heterogeneities were detected among the included studies (I^2^ = 68%, *p* < 0.01). Subgroup analysis, sensitivity analysis, and meta regression analysis were adopted to explore the source of heterogeneity. The random effects model was applied for meta-analysis, which suggested HDL was significantly elevated after exercise intervention compared to that of the control group in patients with NAFLD (WMD = 0.11, 95%CI: 0.05~0.17, Z = 3.82, *p* < 0.01). Subgroup analysis showed that aerobic exercise could significantly increase HDL in patients with NAFLD (WMD = 0.12, 95%CI: 0.05~0.18, Z = 3.56, *p* < 0.01) with high heterogeneities (I^2^ = 70%, *p* < 0.01) (Figure 6). In addition, resistance exercise had little effect in the regulation of HDL in patients with NAFLD with no significant difference (WMD = 0.00, 95%CI: −0.06~0.07, Z = 0.05, *p* > 0.05). No heterogeneity was detected in resistance exercise subgroups (I^2^ = 0.0%, *p* > 0.05) (Figure 6).

The sensitivity analysis suggested a stable finding in each study as the low sensitivity had little impact on the heterogeneity (Figure S4). In the meta regression analysis, although publication year and sample size exhibited slight effects on the heterogeneity, no factor was found to have a significant impact on the heterogeneity of the included studies (*p* > 0.05). Therefore, publication year and sample size might be tfactors affecting the heterogeneity of studies but with no significant difference (Table 5). In addition, publication bias existed in the 13 included studies (t = −2.74, *p* = 0.02).

#### 3.4.5. Meta-Analysis and Publication Bias Evaluation of ALT

**Figure 7:**
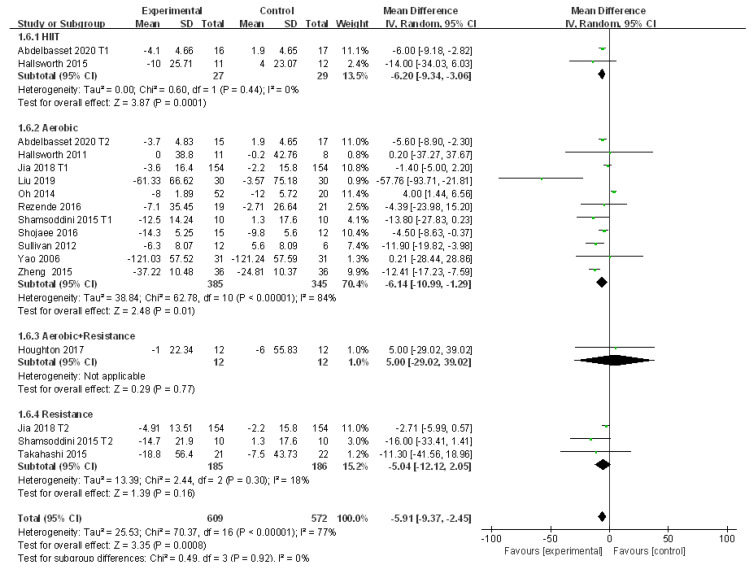
Subgroup analysis of different exercise styles on ALT.

A total of 14 studies reported the change of ALT in patients with NAFLD before and after exercise intervention with significant large heterogeneities (I^2^ = 77%, *p* < 0.01). The source of heterogeneity was analyzed by subgroup analysis, sensitivity analysis, and meta regression analysis. Meta-analysis was conducted using the random effects model, which revealed that the level of ALT in patients with NAFLD was significantly reduced after exercise intervention compared to that of the control group (WMD = −5.91, 95%CI: −9.37~−2.45, Z = 3.35, *p* < 0.01). Subgroup analysis showed that aerobic exercise (WMD = −6.14, 95%CI: −10.99~−1.29, Z = 2.48, *p* < 0.05) and HIIT (WMD = −6.20, 95%CI: −9.34~−3.06, Z = 3.87, *p* < 0.01) could significantly reduce ALT in patients with NAFLD. There was no heterogeneity between the HIIT subgroups (I^2^ = 0.0%, *p* > 0.05), but significant heterogeneity was detected in the aerobic exercise subgroups (I^2^ = 84%, *p* < 0.01) (Figure 7).

The sensitivity analysis showed that excluding several articles had little impact on the heterogeneity (Figure S5). The factor of diet was found to significantly affect the heterogeneity of the included studies (*p* < 0.05) and the other four did not exhibit significant impacts (*p* > 0.05) in the meta regression analysis (Table 6). In addition, Egger’s test revealed that publication bias did not exist in the 14 included studies (t = −1.88, *p* > 0.05).

#### 3.4.6. Meta-Analysis and Publication Bias Evaluation of AST

**Figure 8:**
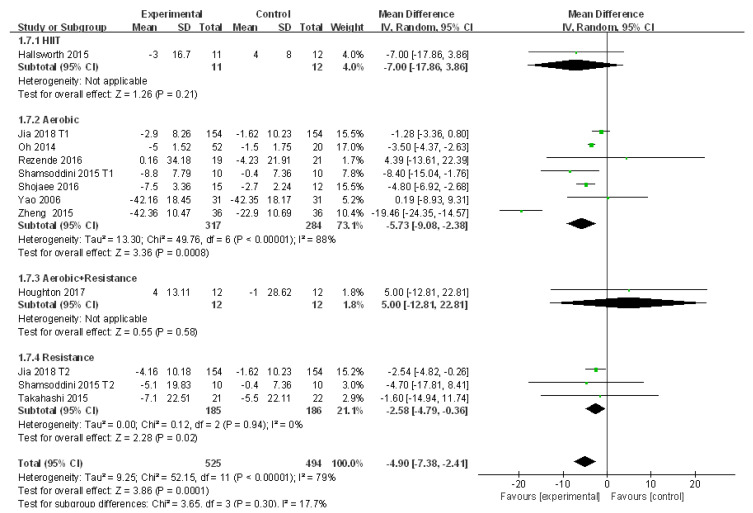
Subgroup analysis of different exercise styles on aspartate aminotransferase (AST).

Ten studies reported the alteration of AST in patients with NAFLD before and after exercise intervention. Large heterogeneities were detected among the included studies (I^2^ = 79%, *p* < 0.01). The random effects model was applied for meta-analysis, and the source of heterogeneity was analyzed by subgroup analysis, sensitivity analysis, and meta regression analysis. The random effects model revealed that AST was significantly decreased after exercise intervention compared to that of the control group in patients with NAFLD (WMD = −4.90, 95%CI: −7.38~−2.41, Z = 3.86, *p* < 0.01). Subgroup analysis revealed that aerobic exercise could significantly reduce AST in patients with NAFLD (WMD = −5.73, 95%CI: −9.08~−2.38, Z = 3.36, *p* < 0.01) with significant heterogeneity detected in aerobic exercise subgroups (I^2^ = 88%, *p* < 0.01). In addition, resistance exercise could significantly decrease AST in patients with NAFLD (WMD = −2.58, 95%CI: −4.79~−0.36, Z = 2.28, *p* < 0.05), and no heterogeneity was detected in resistance exercise subgroups (I^2^ = 0.0%, *p* > 0.05) (Figure 8).

The source of heterogeneity was initially explored by sensitivity analysis, which found that the low sensitivity had little impact on the heterogeneity (Figure S6). In the later meta regression analysis, publication year and medication were found to be key factors affecting the heterogeneity of the included studies (*p* < 0.05), whereas the other three factors did not exhibit significant effects (*p* > 0.05). Therefore, publication year and medication were the main contributors to the heterogeneity. (Table 7). Moreover, no publication bias was found in the 10 included studies (t = −0.54, *p* > 0.05).

#### 3.4.7. Meta-Analysis and Publication Bias Evaluation of GGT

**Figure 9:**
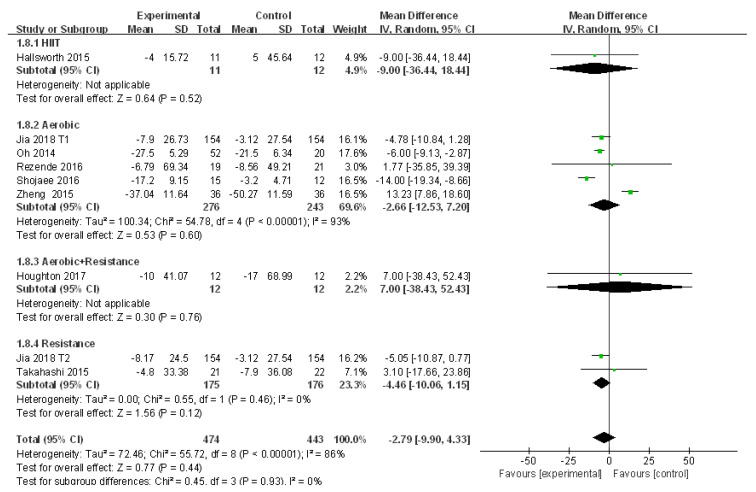
Subgroup analysis of different exercise styles on γ-glutamyl transpeptidase (GGT).

A total of nine studies assessed the efficacy of exercise intervention in the treatment of NAFLD by analyzing the change of GGT with moderate heterogeneities (I^2^ = 86%, *p* < 0.01). Meta-analysis was conducted using the random effects model, and the source of heterogeneity was analyzed by subgroup analysis and sensitivity analysis. The random effects model suggested that exercise intervention tended to decrease GGT in patients with NAFLD but with no significant difference compared to that of the control group (WMD = −2.79, 95%CI: −9.90~4.32, Z = 0.77, *p* > 0.05). Subgroup analysis revealed that aerobic exercise tended to reduce GGT in patients with NAFLD with no significant difference (WMD = −2.66, 95%CI: −12.53~−7.20, Z = 0.53, *p* > 0.05), whereas significant heterogeneity was detected in aerobic exercise subgroups (I^2^ = 93%, *p* < 0.01). In addition, resistance exercise tended to decrease GGT in patients with NAFLD with no significant difference (WMD = −4.46, 95%CI: −10.06~1.15, Z = 1.56, *p* > 0.05) or heterogeneity (I^2^ = 0.0%, *p* > 0.05) (Figure 9).

The sensitivity analysis suggested a valid finding of each study since excluding several articles had a slight impact on the heterogeneity (Figure S7). In addition, given that the number of included studies was less than 10, meta regression and publication bias analyses were not conducted.

#### 3.4.8. Meta-Analysis and Publication Bias Evaluation of BMI

**Figure 10:**
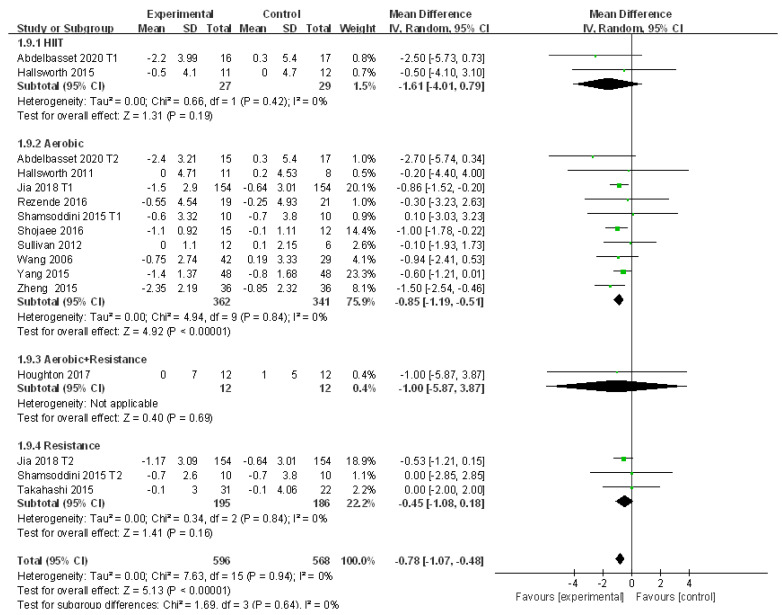
Subgroup analysis of different exercise styles on body mass index (BMI).

Thirteen studies looked at the alteration of BMI in patients with NAFLD who received exercise intervention with no heterogeneity detected (I^2^ = 0%, *p* > 0.05). Therefore, a fixed effects model was adopted for meta-analysis, and the results showed that the level of BMI in patients with NAFLD was significantly reduced after exercise intervention compared to that of the control group (WMD = −0.78, 95%CI: −1.07~−0.48, Z = 5.13, *p* < 0.01). Subgroup analysis showed that aerobic exercise could significantly reduce BMI in patients with NAFLD (WMD = −0.85, 95%CI: −1.19~−0.51, Z = 4.92, *p* < 0.01) with no heterogeneity detected (I^2^ = 0%, *p* > 0.05). In addition, Egger’s test revealed that publication bias did not exist in the 13 included studies (t = −0.12, *p* > 0.05) (Figure 10).

The sensitivity analysis and meta regression analysis were not conducted here because no heterogeneity was detected.

## 4. Discussion

Exercise has many benefits, such as promoting the metabolism of blood lipids, reducing liver fat and improving the quality of life. Various studies have proven that exercise intervention is effective in patients with NAFLD. However, considering individual heterogeneity remains a challenge to developing appropriate exercise prescriptions for patients with NAFLD [29]. As far as we know, this study is the first to analyze the effects of different exercise modes on blood biochemical indexes (TG, TC, LDL, and HDL), liver function related enzymes (ALT, AST, and GGT), and BMI in patients with NAFLD. This meta-analysis showed that aerobic exercise and resistance exercise can significantly improve TG and AST in patients with NAFLD, which is consistent with a recent study [30]. Aerobic exercise and HIIT can significantly reduce ALT in patients with NAFLD. These findings are necessary for people with NAFLD because some types of exercise may lead to sports injuries, and it is important to choose the exercise that suits the patient. For example, for obese patients with NAFLD, some types of aerobic exercise may cause joint pressure or injury, and resistance exercise is more appropriate. In addition, aerobic exercise can significantly improve TC, LDL, HDL, and BMI in patients with NAFLD, while resistance exercise and HIIT do not show similar results in this meta-analysis; most studies were about the effects of aerobic exercise on NAFLD, while the effects of other exercise methods need to be studied further with more randomized trials. Various studies have proven that exercise intervention is effective in patients with NAFLD.

Subgroup analysis, sensitivity analysis, and univariate meta regression analysis were performed to explore the source of heterogeneity and ensure the accuracy of the results [31]. However, subgroup analysis and sensitivity analysis failed to find the source of heterogeneity. The effects of publication year, sample size, intervention time, diet, and drugs on heterogeneity were evaluated by meta regression analysis. The results showed that the published year was the main factor of heterogeneity in the study of TG, TC, and ALT, which indicated that the results for these subjects lacked good consistency in time series, and diet and drugs were the key factors for the source of heterogeneity in the study of LDL and AST, respectively. These meta regression results suggest the reasons for the heterogeneity of the data in our literature, which provides a reference for the follow-up design of randomized controlled trials that should be careful to deal with the factors affecting heterogeneity. For example, when studying AST, we should pay attention to the additional effects of drug factors on it. In addition, an Egger’s test was conducted to evaluate the publication bias of the selected studies, and the results showed that there was no significant publication offset risk except HDL, which ensured the reliability and stability of the research results.

Various clinical trials have confirmed the effectiveness of exercise intervention in the treatment of NAFLD [32,33]. The results of this meta-analysis also show that aerobic exercise can significantly improve liver lipids and liver enzymes in patients with NAFLD. This suggests that aerobic exercise may lead to a decrease in liver fat and fat storage through a calorie consumption mechanism, and may also reduce liver enzymes, which is good for liver health. Previous studies have also shown that aerobic exercise can improve NAFLD by reducing fatty acid synthase and acetyl-CoA carboxylase, increasing liver mitochondrial content and oxidation, activating AMP-dependent protein kinases, reducing fat synthesis, and increasing lipid oxidation. It also provides a scientific basis for aerobic exercise to improve a variety of indicators in patients with NAFLD. In addition, the therapeutic effects of resistance exercise and HIIT on NAFLD need to be verified by more studies.

Our research has several limitations. First of all, some of the included studies did not clearly describe the intensity of sports intervention, so we cannot carry out subgroup analysis by intensity, which may affect the effect of sports activities. Another disadvantage is that although we searched thoroughly for published studies, we cannot rule out the possibility of missing valid unpublished studies, which may explain the significant publication bias of HDL. In addition to the eight indicators included in this study, liver fat and magnetic resonance imaging can also be used as indicators to evaluate the improvement of NAFLD [34]. Further research should include more exercise methods and detection indicators and comprehensively evaluate the efficacy of different exercise methods in the treatment of NAFLD, so as to provide scientific and comprehensive reference for exercise prescription.

## 5. Conclusions

Our meta-analysis included 18 studies to characterize the efficacy of different exercise methods in the treatment of NAFLD by screening the changes of eight indicators of blood biochemical indicators, liver function related enzymes, and BMI. Subgroup analysis revealed the different characteristics of various exercise methods in the treatment of NAFLD. Patients with high levels of TG or AST are recommended to do aerobic or resistance exercises. Aerobic or high-intensity interval trainings are better for patients with a high level of ALT. Aerobic exercise can also be proposed as the first choice for patients with NAFLD to improve the levels of TC, LDL, HDL, or BMI.

## Figures and Tables

**Table 1:** Characteristics of the included trials.

Study	Year	Research Object			Intervention					Diet	Drug	Outcomes
		Participants	Gender (m/f)	Age	Exercise Type	Intensity	Duration (min)	Frequency (n/week)	Duration (week)			
Abdelbasset	2020	T1 16	10/6	54.4 ± 5.8	HIIT	80–85% VO_2max_	40	3	8			①②③④⑤⑥
		T2 15	8/7	54.9 ± 4.7	Aerobic	60–70% VO_2max_	40–50	3	8			
		C 16	9/7	55.2 ± 4.3	No exercise							
Hallsworth	2011	T 11			Aerobic	60% HRM	45–60	3	8			①②③⑥
		C 8			No exercise							
Hallsworth	2015	T 11	11/0	54 ± 10	HIIT		30–40	3	12			①②③⑥⑦⑧
		C 12	12/0	52 ± 12	No exercise							
Houghton	2017	T 12	12/0	54 ± 12	Aerobic + Resistance		45–60	3	12			①②③⑥⑦⑧
		C 12	12/0	51 ± 16	No exercise							
Huang	2014	T 28			Aerobic			5	24	Diet		②③④⑤
		C 28			No exercise							
Jia	2018	T1 154	78/76	54.6 + 7.5	Aerobic	50–70% HRM	45	3	24			①②③④⑤⑥⑦⑧
		T2 154	78/76	55.1 ± 7.4	Resistance							
		C 154	75/79	54.2 + 7.5	No exercise							
Liu	2019	T 30	12/18	60.5 ± 8.5	Aerobic		60	4	16			②③④⑤⑥
		C 30	17/13	61.5 ± 8.2	No exercise							
Oh	2014	T 52	52/0	49.1 ± 1.3	Aerobic	>40% HRM	90	3	12	Diet		②④⑤⑥⑦⑧
		C 20	20/0	53.2 ± 2.1	No exercise							
Rezende	2016	T 19	19/0	56.2 ± 7.8	Aerobic		30–50	2	24			①②④⑤⑥⑦⑧
		C 21	21/0	54.5 ± 8.9	No exercise							
Shah	2009	T 9	2/7	68.5 ± 1.3	Aerobic		90	3	24	Diet		②③④⑤
		C 9	3/6	68.6 ± 1.1	No exercise							
Shamsoddini	2015	T1 10	10/0	39.7 ± 6.3	Aerobic	60% HRM	45	3	8			①⑥⑦
		T2 10	10/0	45.9 ± 7.3	Resistance		45					
		C 10	10/0	45.8 ± 7.3	No exercise							
Shojaee	2016	T 15	15/0	52.4 ± 2.2	Aerobic	40–60% HRM	60	4	16			①②③④⑤⑥⑦⑧
		C 12	12/0	52.8 ± 3.0	No exercise							
Sullivan	2012	T 12	4/8	48.6 ± 2.2	Aerobic	45–55% HRM	30–60	5	16			①②③④⑤⑥
		C 6	1/5	47.5 ± 3.1	No exercise							
Takahashi	2015	T 31	9/22	55.5 ± 13.2	Resistance		20–30	3	12			①②④⑤⑥⑦⑧
		C 32	10/12	51.4 ± 14.8	No exercise							
Wang	2006	T 32	14/28	51.9 ± 7.7	Aerobic		60	3	12		Drug	①②③④⑤
		C 29	11/18	49.2 ± 8.7	No exercise							
Yang	2015	T 48	41/7	47.1 ± 3.9	Aerobic		60	3	24			①②③④⑤
		C 48	42/6	48.4 ± 4.8	No exercise							
Yao	2006	T 31			Aerobic		40	7	12		Drug	②③⑤⑥⑦
		C 31			No exercise							
Zheng	2015	T 36	22/14	42.3 ± 10.3	Aerobic	60–75% HRM	40–90	4	24		Drug	①②③④⑥⑦⑧
		C 36	22/14	43.2 ± 9.5	No exercise							

Note: ① = TG; ② = TC; ③ = LDL; ④ = HDL; ⑤ = ALT; ⑥ = AST; ⑦ = GGT; ⑧ = BMI.

**Table 2:** Meta regression analysis of heterogeneous factors of TG.

Research Factors	Regression Coefficients	95% CI	t	*p*
publication year	−0.066	−0.128~−0.003	−2.29	0.041
sample size	0.001	−0.001~0.004	1.25	0.234
intervention time	−0.009	−0.046~0.028	−0.55	0.596
diet	−0.002	−0.061~0.057	−0.07	0.942
medication	0.031	−0.043~0.104	0.91	0.380

**Table 3:** Meta regression analysis of heterogeneous factors of TC.

Research Factors	Regression Coefficients	95%CI	t	*p*
publication year	−0.046	−0.092~−0.001	−2.28	0.045
sample size	0.002	−0.000~0.003	1.83	0.096
intervention time	−0.006	−0.047~0.035	−0.35	0.735
diet	0.037	−0.044~0.118	1.02	0.330
medication	0.023	−0.027~0.074	1.02	0.332

**Table 4:** Meta regression analysis of heterogeneous factors of LDL.

Research Factors	Regression Coefficients	95%CI	t	*p*
publication year	−0.047	−0.112~−0.019	−1.62	0.139
sample size	0.001	−0.000~0.002	1.40	0.194
intervention time	−0.014	−0.039~0.011	−1.25	0.244
diet	0.091	−0.057~0.124	6.18	0.000
medication	0.021	−0.024~0.066	1.06	0.318

**Table 5:** Meta regression analysis of heterogeneous factors of HDL.

Research Factors	Regression Coefficients	95% CI	t	*p*
publication year	0.024	−0.001~−0.048	2.20	0.055
sample size	−0.001	−0.002~0.000	−2.01	0.076
intervention time	0.007	−0.007~0.022	1.11	0.297
diet	−0.013	−0.033~0.006	−1.54	0.159
medication	0.021	−0.024~0.066	−1.23	0.249

**Table 6:** Meta regression analysis of heterogeneous factors of alanine aminotransferase (ALT).

Research Factors	Regression Coefficients	95% CI	t	*p*
publication year	0.612	−0.754~−1.979	0.99	0.345
sample size	−0.000	−0.043~0.043	0.00	1.000
intervention time	0.319	−0.548~1.186	0.81	0.435
diet	−1.375	−2.172~−0.577	−3.79	0.003
medication	0.962	−0.034~1.959	2.13	0.057

**Table 7:** Meta regression analysis of heterogeneous factors of AST.

Research Factors	Regression Coefficients	95%CI	t	*p*
publication year	−2.941	−5.084~−0.798	−3.36	0.015
sample size	0.013	−0.014~0.040	1.19	0.278
intervention time	0.633	−0.428~1.693	1.46	0.195
diet	0.307	−0.141~−0.754	1.68	0.144
medication	2.574	−1.162~3.987	4.46	0.004

## Data Availability

Not applicable.

## Supplementary Materials

The following are available online at https://www.mdpi.com/1660-4601/18/6/3242/s1, Figure S1: Sensitivity analysis of the literature on TG, Figure S2: Sensitivity analysis of the literature on TC, Figure S3: Sensitivity analysis of the literature on LDL, Figure S4: Sensitivity analysis of the literature on HDL, Figure S5: Sensitivity analysis of the literature on ALT, Figure S6: Sensitivity analysis of the literature on AST, Figure S7: Sensitivity analysis of the literature on GGT.
