# Supplementary material for: Effect of Different Exercise Methods on Non-Alcoholic Fatty Liver Disease: A Meta-Analysis and Meta-Regression
Source: Int J Environ Res Public Health. 2021 Mar 21;18(6):3242. doi: 10.3390/ijerph18063242 (PMC8004001; doi:10.3390/ijerph18063242)
Supplement: Supplementary file 1 [file ijerph-18-03242-s001.pdf]

Supplementary Materials

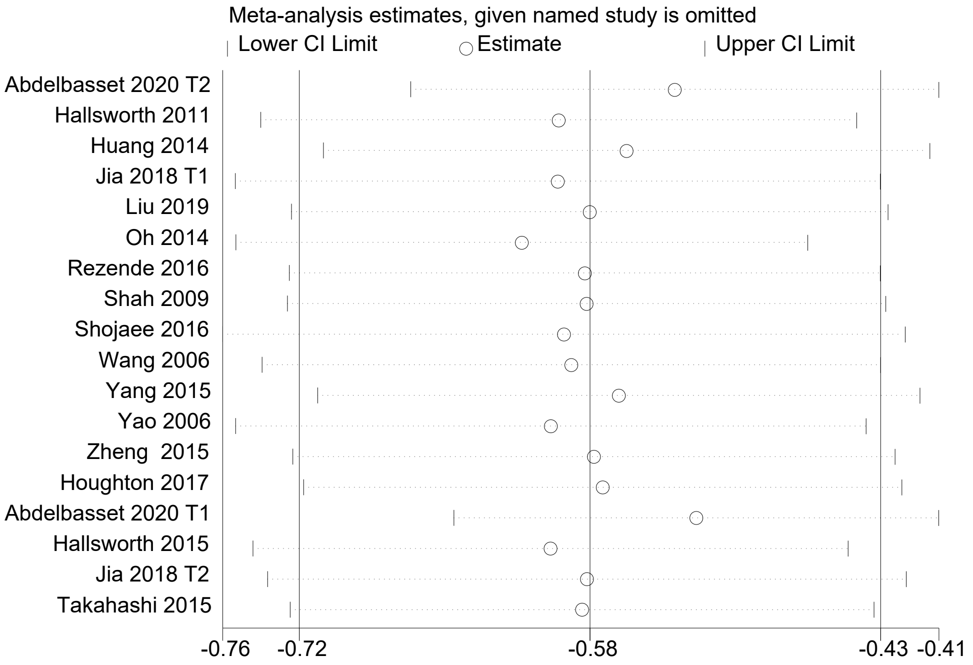

Figure S1. Sensitivity analysis of the literatures on TG.

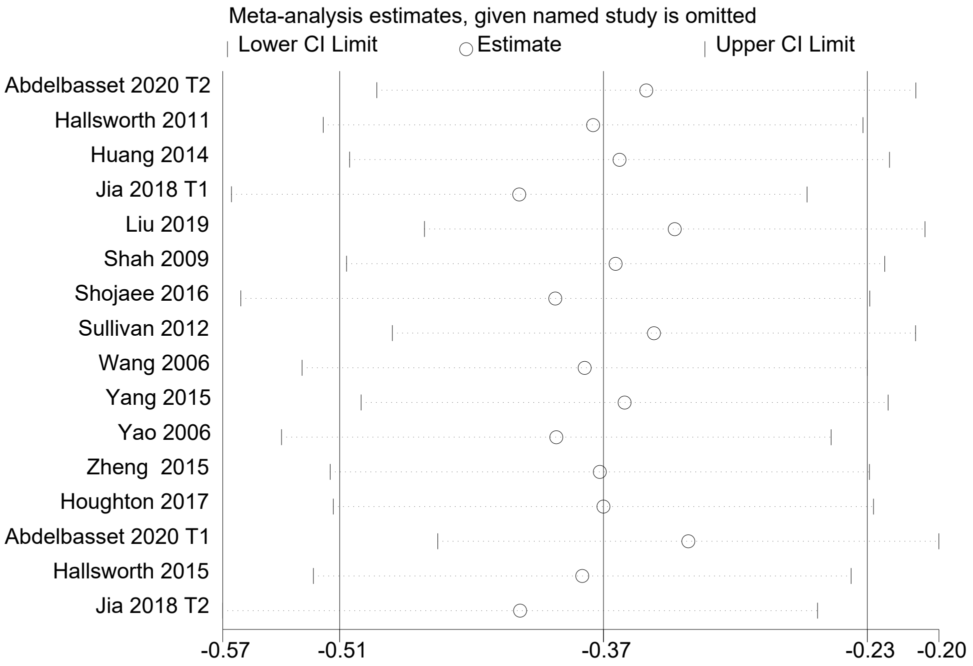

Figure S2. Sensitivity analysis of the literatures on TC.

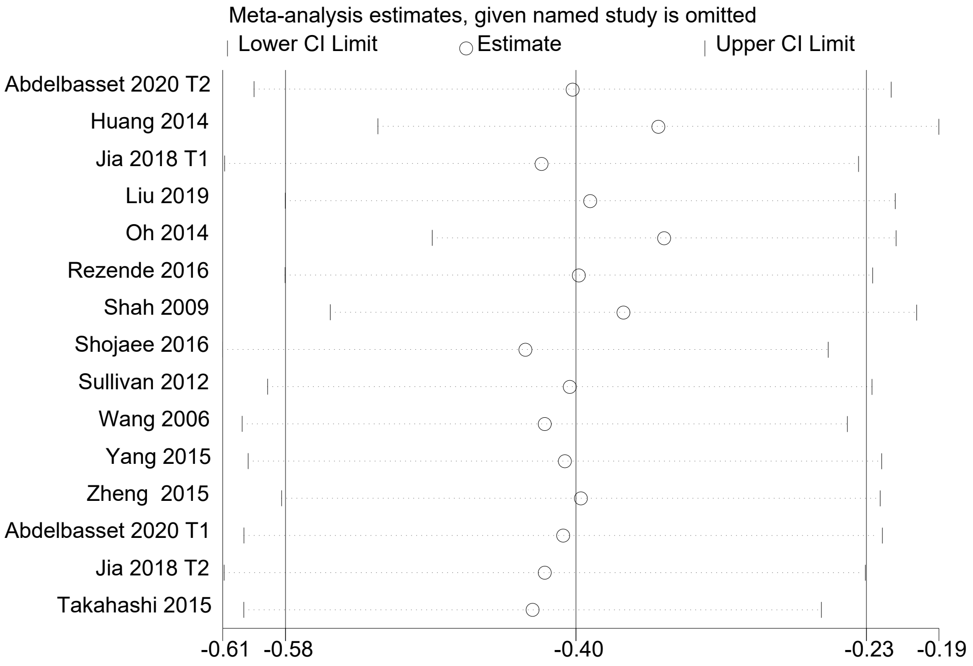

Figure S3. Sensitivity analysis of the literatures on LDL.

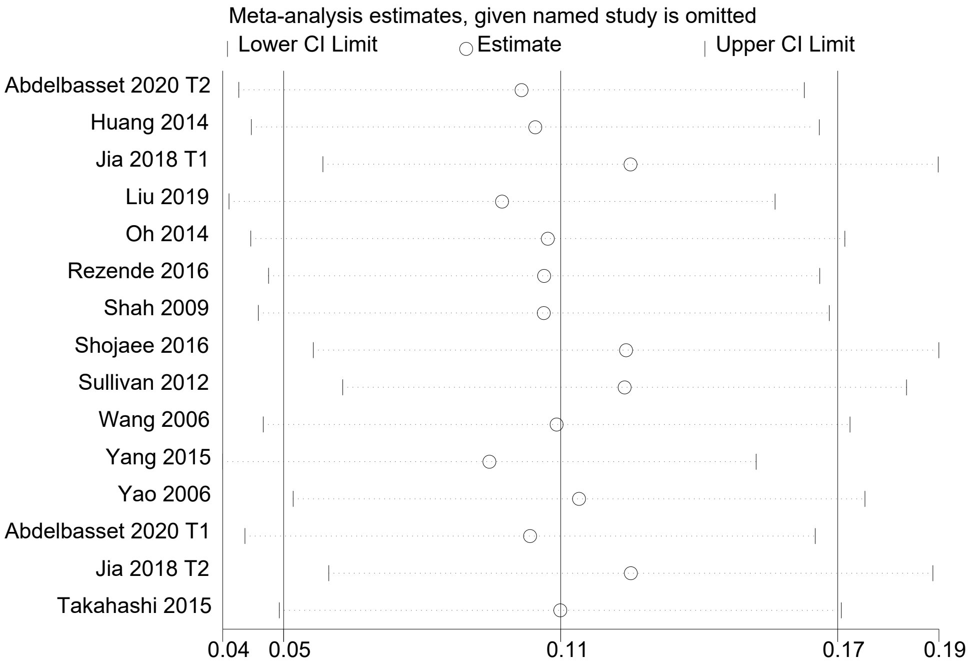

Figure S4. Sensitivity analysis of the literatures on HDL.

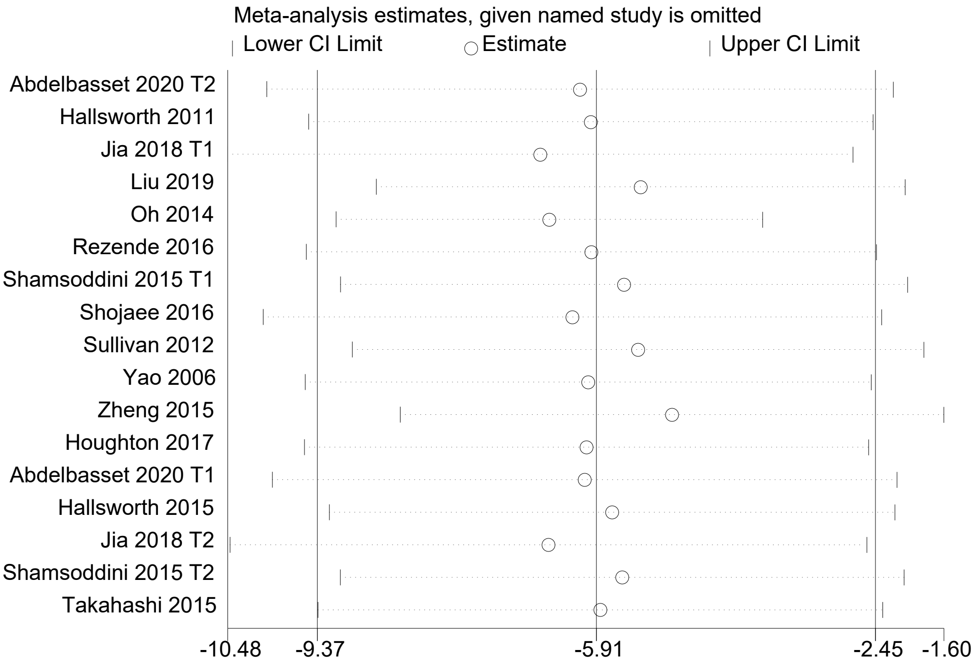

Figure S5. Sensitivity analysis of the literatures on ALT.

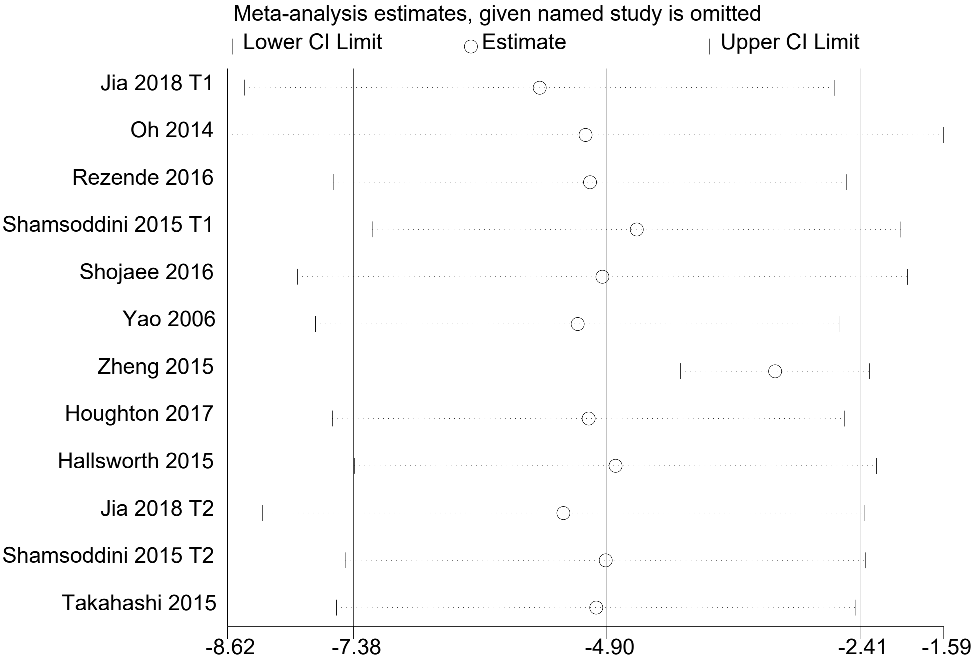

Figure S6. Sensitivity analysis of the literatures on AST.

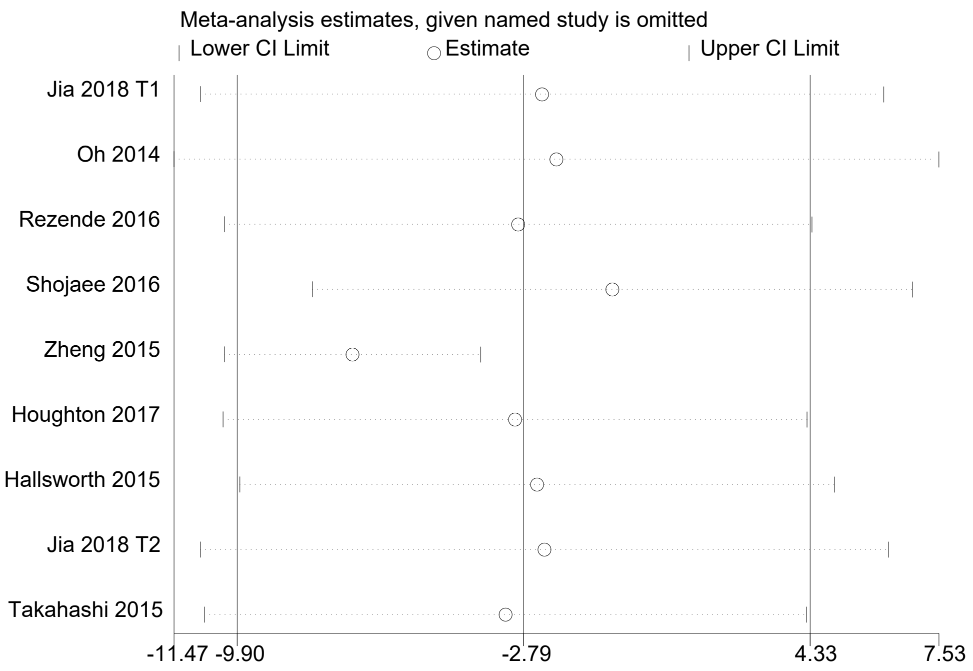

Figure S7. Sensitivity analysis of the literatures on GGT.
